# State Policies on Access to Vaccination Services for Low-Income Adults

**DOI:** 10.1001/jamanetworkopen.2020.3316

**Published:** 2020-04-27

**Authors:** Charleigh J. Granade, Russell F. McCord, Alexandra A. Bhatti, Megan C. Lindley

**Affiliations:** 1 Immunization Services Division, National Center for Immunization and Respiratory Diseases, Centers for Disease Control and Prevention, Atlanta, Georgia; 2 Oak Ridge Institute for Science and Education, Department of Energy, Washington, DC; 3 Now with IHRC Inc, Atlanta, Georgia; 4 Public Health Law Program, Center for State, Tribal, Local, and Territorial Support, Centers for Disease Control and Prevention, Atlanta, Georgia; 5 Cherokee Nation Assurance, Arlington, Virginia; 6 Now with Global Policy, Communications & Population Health, Merck & Co Inc, North Wales, Pennsylvania

## Abstract

**Importance:**

State vaccination benefits coverage and access for adult Medicaid beneficiaries vary substantially. Multiple studies have documented lower vaccination uptake in publicly insured adults compared with privately insured adults.

**Objective:**

To evaluate adult Medicaid beneficiaries’ access to adult immunization services through review of vaccination benefits coverage in Medicaid programs across the 50 states and the District of Columbia.

**Design, Setting, and Participants:**

A public domain document review with supplemental semistructured telephone survey was conducted between June 1, 2018, and June 14, 2019, to evaluate vaccination services benefits in fee-for-service and managed care organization arrangements for adult Medicaid beneficiaries in the 50 states and the District of Columbia (total, 51 Medicaid programs).

**Exposures:**

Document review of benefits coverage for adult immunization services and supplemental survey with validation of document review findings.

**Main Outcomes and Measures:**

Benefits coverage for adult Medicaid beneficiaries and reimbursement amounts for vaccine purchase and administration.

**Results:**

Public domain document review was completed for all 51 jurisdictions. Among these, 44 Medicaid programs (86%) validated document review findings and completed the survey. Only 22 Medicaid programs (43%) covered all 13 Advisory Committee on Immunization Practices–recommended adult immunizations under both fee-for-service and managed care organization arrangements. Most fee-for-service arrangements (37 of 49) reimbursed health care professionals using any of the 4 approved vaccine administration codes; however, 8 of 49 programs did not separately reimburse for vaccine administration to adult Medicaid beneficiaries. Depending on administration route, median reimbursement for adult vaccine administration ranged from $9.81 to $13.98 per dose. Median per-dose reimbursement for adult vaccine purchase was highest for 9-valent human papillomavirus vaccine ($204.87) and lowest for *Haemophilus influenzae* type b vaccine ($18.09). Median reimbursement was below the private sector price for 7 of the 13 included vaccines.

**Conclusions and Relevance:**

Even in programs with complete vaccination benefits coverage, reimbursement amounts to health care professionals for vaccine purchase and administration may not fully cover vaccination provision costs. Reimbursement amounts below costs may reduce incentives for health care professionals to vaccinate low-income adults and thereby limit Medicaid adult beneficiary access to vaccination.

## Key Points

**Question:**

What level of access to vaccination services do Medicaid programs provide to adult beneficiaries enrolled in fee-for-service and managed care organization arrangements?

**Findings:**

In this survey study of Medicaid programs, 22 of 51 programs covered all 13 adult vaccines recommended by the Advisory Committee on Immunization Practices for both fee-for-service and managed care organization enrollees. Reimbursement for vaccine administration was disparate; median vaccine purchase reimbursement was highly variable relative to manufacturer-reported private sector price.

**Meaning:**

These findings suggest that most adult Medicaid beneficiaries do not have access to all 13 Advisory Committee on Immunization Practices–recommended adult vaccines; low reimbursement for vaccine administration and purchase may disincentivize health care professionals to vaccinate low-income adults.

## Teaser

This survey study evaluates adult Medicaid beneficiaries’ access to adult immunization services through review of vaccination benefits coverage in Medicaid programs across the 50 states and the District of Columbia.

## Introduction

Medicaid traditionally provides health insurance to low-income children and parents, pregnant women, the elderly, and disabled individuals at little to no cost. In 2010, the Patient Protection and Affordable Care Act extended Medicaid eligibility to include childless adults with incomes up to 138% of the federal poverty level.^1^ Enrollment rapidly increased; by 2017, adults enrolling under this expansion made up 19.4% of the total Medicaid population.^2^ Medicaid currently provides health insurance to an estimated 37.5 million adults across all eligibility groups in the United States.^3^

For the poorest citizens in the United States, Medicaid is the primary source of funding for health-related services, including vaccinations. Research suggests that individuals with health insurance have higher receipt than uninsured individuals of preventive services,^4,5,6^ but adults with public insurance generally have lower vaccination coverage than do privately insured individuals.^7,8,9,10^ Low adult immunization coverage can burden the US health care system: in 2015, vaccine-preventable diseases in adults cost the United States $9 billion in health care costs and lost productivity.^11^ Pneumonia and influenza are among the top causes of death for US adults, accounting for 55 672 deaths (2% of total deaths) in 2017.^12^ Knowledge regarding current adult immunization policy within Medicaid is limited.^13,14,15^

Adult vaccination services are not a federally mandated benefit for traditionally eligible Medicaid beneficiaries and are therefore determined by individual states. By contrast, benefits packages for adults who enrolled under the Medicaid expansion are required to cover 10 “essential health benefits,” including adult immunization services, with no cost sharing.^16^ Although the Medicaid expansion unified benefits among newly eligible adults, it did not address vaccination benefits for traditionally eligible adults. Copayments are a known barrier to receipt of health services such as vaccination, particularly for low-income populations.^17,18^ Medicaid programs were encouraged to limit copayments through the Section 4106 incentive, in which states received a 1% increase in the Federal Medical Assistance Percentage if their state matched preventive care benefits for adults who enrolled in Medicaid under the expansion and traditionally eligible Medicaid populations with no cost-sharing.^19^ Not all expansion states chose to leverage this increased Federal Medical Assistance Percentage incentive, resulting in persistent financial barriers for some traditionally eligible beneficiaries and potentially solidifying disparities in access between eligibility groups.

We evaluated adult Medicaid beneficiaries’ access to immunization services through review of vaccination benefits coverage in Medicaid programs across the 50 states and the District of Columbia. Specifically, this study examined benefits coverage and reimbursement amounts for vaccine purchase and administration as well as copayment practices for adult Medicaid beneficiaries for each vaccine recommended for adults in 2018 by the Advisory Committee on Immunization Practices (ACIP).^20^

## Methods

The study was completed between June 1, 2018, and June 14, 2019. We conducted a public document review and developed and administered a survey assessing adult immunization policies for Medicaid fee-for-service (FFS) and managed care organization (MCO) arrangements. For the survey, respondent consent was assumed if the Medicaid director or their designee agreed to participate in the survey. To allow for transcription of responses collected via the telephone survey, respondents were asked to verbally consent to being audio recorded. The Centers for Disease Control and Prevention (CDC) determined this study to be research not involving human participants; therefore, it did not require institutional review board approval.

### Document Review

Public domain document review was conducted from April 2, 2018, to April 30, 2019. Using a standard search engine, information related to benefits coverage of, payment for, and copayments for Medicaid adult vaccination services was collected using the following search strings: “[X] state Medicaid plan,” “FFS [fee-for-service] fee schedule,” and “provider manuals Medicaid.”

Public domain review materials were organized into a brief document and integrated into the survey tool. The document comprised Medicaid program population estimates, expansion status, Section 4106 status, income eligibility limits, distribution of FFS and MCO arrangements, federally qualified health center benefits, and FFS reimbursement fee schedules.

### Survey Design and Administration

The survey was developed by the the CDC’s Public Health Law Program and Immunization Services Division. Using the National Association of Medicaid Directors online directory,^21^ we identified each jurisdiction’s Medicaid program director, who was sent an introductory email along with a summary of the public domain document review for their program and the survey instrument. Up to 5 additional emails were sent to nonresponding programs. Respondents included Medicaid directors and their designated representatives.

Respondents were asked to validate the public domain document review results for their program and to complete a semistructured survey. Both semistructured telephone surveys and written responses were collected from participating programs. The survey questions and probing used in both collection methods were identical, with response method determined by respondent preference. The survey and validation of the public domain document review was conducted from June 1, 2018, to June 14, 2019.

### Adult Vaccination Access and Reimbursement

To understand access to and reimbursement for adult immunization services at the Medicaid program level, we assessed coverage benefits for the following 2018 ACIP-recommended adult immunizations for persons 19 years or older^20^: influenza; tetanus toxoid, reduced diphtheria toxoid, and acellular pertussis (Tdap); measles, mumps, and rubella (MMR); varicella; recombinant zoster; 9-valent human papilloma virus (9vHPV); pneumococcal conjugate; pneumococcal polysaccharide (PPSV23); hepatitis A; hepatitis B; serogroup A, C, W, and Y meningococcal; serogroup B meningococcal; and *Haemophilus influenzae* type b (Hib) vaccines.

Thirty-one *Current Procedural Terminology* (*CPT*) codes for adult vaccines were evaluated using each program’s FFS fee schedule to determine adult vaccination benefits coverage; however, because numerous influenza vaccines are available on the market, only the most commonly covered inactivated influenza vaccine (code 90656), the live attenuated vaccine (code 90672), and the recombinant vaccine (code 90682) were used to assess benefits coverage. In addition, reimbursement to health care professionals for adult vaccine administration was evaluated for *CPT* codes 90471 to 90474, with each administration code dependent on method of delivery (injected vs intranasal) and number of vaccine doses administered during a health care visit.^22^ Median and range of vaccine purchase reimbursement amounts for the most commonly reimbursed *CPT* codes for each ACIP-recommended adult immunization were calculated using available FFS fee schedules and compared with publicly available CDC vaccine prices and manufacturer-reported private sector vaccine prices.^23^ Reimbursement amounts for adult vaccine purchase greater than 1.5 times the interquartile range were noted as outliers.

Medicaid programs were considered to provide coverage benefits consistent with the 2018 ACIP recommendations for adult immunization if their FFS fee schedule demonstrated reimbursement for any *CPT* code for each of the 13 immunizations examined, with the exception of influenza vaccine, for which reimbursement for any of the 3 codes noted above was counted. To supplement document review findings, survey responses from participating Medicaid programs regarding adult vaccination coverage benefits under MCO arrangements, use of copayments for vaccination services under FFS and MCO arrangements, and factors associated with program decisions to cover ACIP-recommended adult immunizations were analyzed.

## Results

Health care professional FFS reimbursement fee schedules were evaluated for 49 of 51 Medicaid programs; the remaining 2 programs, in Hawaii and Tennessee, are both under 100% MCO arrangements. In addition, 44 Medicaid programs (86%) validated public domain document review findings and completed the survey—34 (77%) via telephone and 10 (23%) in writing—and were included in analyses.

### Medicaid Program Coverage of ACIP-Recommended Adult Vaccines

Although most Medicaid programs provided some level of coverage for adult vaccines, only 22 Medicaid programs (43%) covered all 13 ACIP-recommended adult immunizations under both FFS and MCO arrangements (eTable 1 in the Supplement). Although 3 additional programs (in Arkansas, Iowa, and Utah) covered all ACIP-recommended vaccines under FFS, no information regarding their MCO vaccine benefits coverage was available, as these programs did not participate in the survey.

### FFS Arrangements

**Figure 1.**
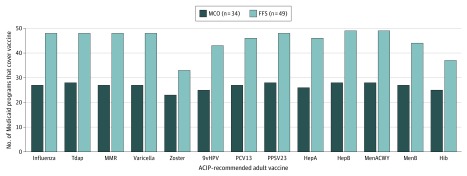
Medicaid Program Coverage of All Advisory Committee on Immunization Practices (ACIP)–Recommended Adult Vaccines by Fee-for-Service (FFS) and Managed Care Organization (MCO) Arrangement Data are from public domain document review and survey. For ACIP-recommended adult immunizations with multiple *Current Procedural Terminology* (*CPT*) codes available, benefits coverage was assumed if any 1 of the associated *CPT* codes was reported as covered. If a Medicaid program covered at least 1 of 3 selected influenza vaccines (inactivated, live attenuated, or recombinant), the program was considered to cover influenza vaccines. 9vHPV indicates 9-valent human papillomavirus vaccine; HepA, hepatitis A vaccine; HepB, hepatitis B vaccine; Hib, *Haemophilus influenzae* type b vaccine; MenACWY, serogroup A, C, W, and Y meningococcal vaccine; MenB, serogroup B meningococcal vaccine; MMR, measles, mumps, and rubella vaccine; PCV13, pneumococcal conjugate vaccine; PPSV23, pneumococcal polysaccharide vaccine; and Tdap, tetanus toxoid, reduced diphtheria toxoid, and acellular pertussis vaccine.

Most Medicaid program FFS arrangements provided adult vaccination benefits coverage for all or most ACIP-recommended adult immunizations (Figure 1). Twenty-four of 49 FFS arrangements provided coverage for all ACIP-recommended adult immunizations. Forty-eight FFS arrangements covered 1 or more influenza vaccines in addition to Tdap, MMR, varicella, and PPSV23. In contrast, benefits coverage was less common for the 9vHPV vaccine (43 of 49 FFS arrangements), Hib vaccine (37 of 49 FFS arrangements), and zoster vaccine (33 of 49 FFS arrangements).

### MCO Arrangements

Thirty-nine of 51 Medicaid programs (76%) covered some proportion of their beneficiaries via MCO arrangements. Because information regarding adult vaccination benefits coverage by individual *CPT* code for MCO arrangements was unavailable, coverage of the ACIP-recommended adult immunization schedule was evaluated using survey responses from 34 of 39 participating programs. Although adult vaccination benefits coverage varied by individual MCO, 21 programs provided coverage for all ACIP-recommended immunizations. Similar to FFS arrangements, coverage for the 9vHPV vaccine (25 of 34 participating programs), Hib vaccine (25 of 34 participating programs), and zoster vaccine (23 of 34 participating programs) was lower than for other vaccines under MCO arrangements (Figure 1).

### Reimbursement to Health Care Professionals for Vaccine Administration

Reimbursement to health care professionals for administration of adult vaccines to FFS beneficiaries varied for each Medicaid program (Table). For the first dose of injected vaccine administered during a visit (*CPT* code 90471), the median reimbursement was $13.62 (range, $3.72 in South Carolina to $28.18 in Alaska); median reimbursement for first intranasal administration (*CPT* code 90473) was $13.98 (range, $3.00 in Michigan to $28.18 in Alaska). Median reimbursement for each subsequent dose of injected vaccine (*CPT* code 90472) was $9.81 and for each subsequent dose of intranasal vaccine (*CPT* code 90474) was $9.92, ranging from $2.00 in New York to $20.80 in New Mexico.

**Table.** Reimbursement Amounts to Health Care Professionals for Adult Vaccine Administration Under FFS Arrangements, by Medicaid Program^a^

State program	Reimbursement amount for vaccine administration, $			
	*CPT* code 90471^b^	*CPT* code 90472^c^	*CPT* code 90473^d^	*CPT* code 90474^e^
Alabama	5.00	NC	NC	NC
Alaska	28.18	18.11	28.18	18.11
Arizona	22.32	11.17	22.32	11.17
Arkansas	NC	NC	NC	NC
California	4.46	NC	NC	NC
Colorado	19.12	11.10	19.12	11.10
Connecticut	NC	NC	NC	NC
Delaware	NC	NC	NC	NC
District of Columbia	15.56	11.83	15.56	11.83
Florida^f^	10.00	10.00	10.00	10.00
Georgia	NC	NC	NC	NC
Hawaii	100%^g^	100%^g^	100%^g^	100%^g^
Idaho	19.29	12.08	19.29	12.08
Illinois	NC	NC	NC	NC
Indiana	17.61	8.90	17.61	8.90
Iowa	5.09	5.09	12.88	6.86
Kansas	14.15	14.15	14.15	14.15
Kentucky	15.60	9.80	15.60	9.80
Louisiana	14.70	9.13	10.43	9.13
Maine	13.43	6.84	8.93	5.94
Maryland	NC	NC	NC	NC
Massachusetts	20.45	9.82	20.45	9.84
Michigan	7.00	7.00	3.00	3.00
Minnesota	12.83	9.76	12.83	9.76
Mississippi	16.90	10.62	16.90	10.62
Missouri	13.01	6.59	8.27	5.65
Montana	21.32	14.08	21.32	14.08
Nebraska	5.80	5.80	5.80	5.80
Nevada	22.22	11.01	22.22	11.01
New Hampshire	5.00	3.05	5.00	3.00
New Jersey	11.50	11.50	NC	NC
New Mexico	20.80	20.80	20.80	20.80
New York	13.23	2.00	8.57	2.00
North Carolina	20.45	20.45	20.45	20.45
North Dakota	15.49	15.49	15.49	15.49
Ohio	19.35	9.50	19.35	9.50
Oklahoma	18.67	11.76	18.67	11.76
Oregon	11.73	8.99	11.73	8.99
Pennsylvania^h^	10.00	10.00	10.00	10.00
Rhode Island	8.16	3.68	8.16	3.68
South Carolina	3.72	3.72	3.72	3.72
South Dakota	9.78	9.78	9.78	10.32
Tennessee	100%^g^	100%^g^	100%^g^	100%^g^
Texas	7.84	7.84	NC	NC
Utah	13.81	13.81	13.81	13.81
Vermont	16.71	10.31	16.71	10.31
Virginia	NC	NC	NC	NC
Washington	12.11	7.46	12.11	7.46
West Virginia	NC	NC	NC	NC
Wisconsin	Bundled^i^	Bundled^i^	Bundled^i^	Bundled^i^
Wyoming	9.67	9.67	9.67	9.67
Median	13.62	9.81	13.98	9.92
Total No. of states	41	39	37	37

Abbreviations: *CPT*, *Current Procedural Terminology*; FFS, fee-for-service; NC, not covered.

^a^ Data are from public domain document review, with presented reimbursement amounts to health care professionals in 2018 US dollars. Because the following Medicaid programs did not participate in the semistructured survey, data from Arkansas, Iowa, New Jersey, North Carolina, and Utah were not validated.

^b^ Including percutaneous, intradermal, subcutaneous, or intramuscular injections; first vaccine administered (single or combination vaccine or toxoid).

^c^ Including percutaneous, intradermal, subcutaneous, or intramuscular injections; each additional vaccine administered (single or combination vaccine or toxoid).

^d^ Immunization administration by intranasal or oral route; first vaccine administered (single or combination vaccine or toxoid).

^e^ Immunization administration by intranasal or oral route; each additional vaccine administered (single or combination vaccine or toxoid).

^f^ The Florida Medicaid program does not provide separate reimbursement for vaccine administration if the vaccine is received through pharmacy services for individuals living in residential facilities.

^g^ Both the Hawaii and Tennessee Medicaid programs were under 100% managed care organization arrangements.

^h^ The Pennsylvania Medicaid program did not reimburse vaccine administration using *CPT* codes 90471 to 90474. Instead, health care professionals received a $10.00 administration fee in conjunction with the national drug code to pay for the actual dose administered.

^i^ The Wisconsin Medicaid program did not use vaccine administration *CPT* codes 90471 to 90474 for reimbursement to health care professionals. Instead, the fee for the vaccine administration was added to the reimbursement for the vaccine and paid as a bundled fee.

Most FFS arrangements (37 of 49) reimbursed health care professionals for adult vaccine administration using any of the 4 approved vaccine administration codes (Table). Eight programs (in Arkansas, Connecticut, Delaware, Georgia, Illinois, Maryland, Virginia, and West Virginia) did not provide separate reimbursement for vaccine administration to adult Medicaid beneficiaries.

Eleven programs granted the same reimbursement for all 4 vaccine administration codes, ranging from $3.72 in South Carolina to $20.80 in New Mexico. The programs with the highest reimbursement amounts to health care professionals under FFS arrangements for a single injected vaccine administration (*CPT* code 90471) were Alaska ($28.18), Arizona ($22.32), and Nevada ($22.22). The programs with the lowest reimbursement amounts to health care professionals were South Carolina ($3.72), California ($4.46), Alabama ($5.00), and New Hampshire ($5.00).

### Reimbursement to Health Care Professionals for Vaccine Purchase

**Figure 2.**
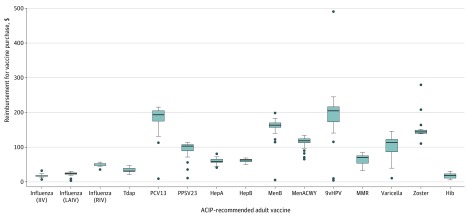
Median and Range of Reimbursement for Advisory Committee on Immunization Practices (ACIP)–Recommended Adult Vaccine Purchase Under Fee-for-Service Arrangements, by *Current Procedural Terminology* (*CPT*) Code Data are from public domain document review. As there are multiple vaccines available for Hep B, MenACWY, MenB, and Hib, medians were calculated only for the most frequently available *CPT* code for each immunization. For each box plot, the middle line designates the median reimbursement amount to health care professionals for vaccine purchase of each of the selected ACIP-recommended adult vaccinations, boxes indicate the interquartile range, and whiskers are drawn to 1.5 × interquartile range. Outliers are represented using circles. 9vHPV indicates 9-valent human papillomavirus vaccine; HepA, hepatitis A vaccine; HepB, hepatitis B vaccine; Hib, *Haemophilus influenzae* type b vaccine; MenACWY, serogroup A, C, W, and Y meningococcal vaccine; MenB, serogroup B meningococcal vaccine; MMR, measles, mumps, and rubella vaccine; PCV13, pneumococcal conjugate vaccine; PPSV23, pneumococcal polysaccharide vaccine; and Tdap, tetanus toxoid, reduced diphtheria toxoid, and acellular pertussis vaccine. Vaccine *CPT* codes are as follows: influenza, inactivated (IIV), 90656; influenza, live attenuated (LAIV), 90682; influenza, recombinant (RIV), 90672; Tdap, 90715; PCV13, 90670; PPSV23, 90732; HepA, 90632; HepB, 90746; MenB, 90620; MenACWY, 90734; 9vHPV, 90651; MMR, 90707; varicella, 90716; zoster, 90750; and Hib, 90648.

Information on reimbursement to health care professionals for vaccine purchase under FFS arrangements was available for 46 of 49 programs. Specific reimbursement amounts for West Virginia, Pennsylvania, and New York were not available, as the respective FFS schedule for each Medicaid program defined reimbursement as one of the following: “carrier-priced” (West Virginia), “national drug code” (Pennsylvania), and “manually priced” (New York). Among all ACIP-recommended adult immunizations, median reimbursement was highest for the 9vHPV vaccine (*CPT* code 90651), at $204.87 (Figure 2). The 9vHPV vaccine also demonstrated the largest per-dose reimbursement range, from $5.27 in Missouri to $491.38 in Mississippi. The ACIP-recommended adult vaccine with the lowest median reimbursement ($18.09) was the Hib vaccine (*CPT* code 90648), ranging from $5.27 in Missouri to $30.80 in Wisconsin.

Median reimbursement for the hepatitis A vaccine (*CPT* code 90632) was $58.65, ranging from $41.11 in the District of Columbia to $80.95 in New Jersey; that for the hepatitis B vaccine (*CPT* code 90746) was $63.30, ranging from $50.64 in the District of Columbia to $69.58 in California (eTable 2 in the Supplement). Median reimbursement was below the private sector price reported by manufacturers to the CDC for 7 of 13 ACIP-recommended adult vaccines, with the largest observed disparities for the varicella, 9vHPV, and Tdap vaccines (eTable 2 in the Supplement).

### Factors Associated With ACIP-Recommended Adult Vaccination Benefits

Survey respondents were asked to describe factors associated with their program’s decision to cover ACIP-recommended adult immunizations. Respondents most commonly cited ACIP and CDC recommendations as the most important factor (38 of 44), followed by state and local health professional recommendations (18 of 44). Respondents also cited state health agency recommendations (10 of 44), public attention (8 of 44), and legislative interest (7 of 44). Other reasons for deciding to cover an ACIP-recommended adult vaccine included US Food and Drug Administration approval, “because it is the right thing to do,” and as a result of infectious disease outbreaks. In Oregon, respondents indicated that their Medicaid program collaborated with a health evidence committee when deciding whether to cover an ACIP-recommended adult immunization.

### Copayments for Adult Vaccination Services

Among the Medicaid programs surveyed, 12 of 44 confirmed that their jurisdiction implemented the Section 4106 incentive and therefore covered all ACIP-recommended adult immunizations with no cost sharing. An additional 17 of 44 programs prohibited cost sharing for immunization services for adult Medicaid beneficiaries without use of the incentive. In the 15 remaining programs in which cost sharing is present, 14 permitted copayments for traditionally eligible adults who receive vaccination benefits under FFS arrangements. Specifically, copayments were permitted for vaccines received in the pharmacy setting (Maryland and Indiana) and for persons older than 21 years who are not pregnant (Oklahoma). In programs permitting copayments for FFS beneficiaries, copayments were prohibited for some populations, including pregnant women (8 of 14), nursing home residents (6 of 14), and individuals under hospice care (3 of 14).

Although most Medicaid programs prohibited MCO arrangements from establishing copayments for adult vaccination, MCOs within 6 of 15 programs permitted copayments for receipt of immunization services. In Florida, copayments were permitted for adult vaccination and are determined by vaccine type. In Georgia, MCO arrangements did not restrict copayments for adult immunization services; therefore, all adult Medicaid beneficiaries are subject to copayments. Although Minnesota, Pennsylvania, Maryland, and Indiana are all expansion states, copayments for adult vaccination were permitted for certain beneficiary populations. However, copayments were prohibited for the following populations: pregnant women, individuals receiving hospice care, and nursing home residents (Minnesota and Pennsylvania); adults receiving vaccinations in the primary care setting (Maryland and Pennsylvania); Native American Indians (Minnesota); and recipients of breast and cervical cancer treatments (Pennsylvania). Cost-sharing policies for MCO arrangements in Indiana were unavailable.

## Discussion

This study found that most Medicaid programs provided some level of reimbursement for adult vaccine administration and purchase. However, only 22 of 51 programs covered all 2018 ACIP-recommended adult immunizations for both FFS and MCO beneficiaries; of those, only 14 provided vaccination benefits without copayments. Adult vaccination coverage and access varied between FFS and MCO arrangements within Medicaid programs. Inequities regarding access to adult immunization services are evident, and incomplete vaccination benefits coverage across Medicaid programs are likely associated with fewer Medicaid beneficiaries receiving the recommended vaccines.

Stewart and colleagues^14^ conducted a public document review and survey of Medicaid directors evaluating adult immunization policy under FFS arrangements, comparing benefits coverage with the 2012 ACIP-recommended adult immunization schedule, and found that 36 of 51 Medicaid FFS arrangements covered all recommended adult vaccines. In the present study, only 24 of 49 FFS arrangements and 21 of 34 MCO arrangements covered the 2018 ACIP-recommended adult immunization schedule. The number of routinely recommended adult vaccines has increased since 2012 from 10 to 13. Furthermore, the 9vHPV vaccine costs more per dose than the HPV vaccines used in 2012,^23^ and recombinant zoster vaccine recipients require 2 doses for complete vaccination,^20^ resulting in higher program costs to cover these vaccines. Among Medicaid programs that do not cover all ACIP-recommended adult immunizations, most do not have full coverage as a result of not covering the 9vHPV vaccine, recombinant zoster vaccine, or both, consistent with previous studies.^14^

Historically, Medicaid reimbursement rates to health care professionals are significantly below Medicare or private sector rates for both vaccine purchase and administration.^24,25,26^ A 2014 survey of family and general internal medicine physicians found that 55% of respondents thought they lost money administering vaccines to adult Medicaid beneficiaries, while 25% or fewer of respondents thought they lost money administering vaccines to adults covered by other public and private payers.^25^ In our study, the median reimbursement amount to health care professionals for administration of a single adult vaccination via injection was $13.62 and for intranasal administration of a single adult vaccination was $13.98, falling within previously reported reimbursement ranges. These median reimbursements are below per-dose costs to administer vaccines to adults estimated by a recent study ($15-$23, depending on type of health care professional).^27^ Similarly, median reimbursement for vaccine purchase was below manufacturer-reported private sector costs for 7 of 13 immunizations examined in this study. Although vaccine purchase prices for individual health care professionals are negotiated with manufacturers or distributors,^25^ our findings regarding median reimbursement and the wide variation among programs suggest that Medicaid payments for adult vaccination might fail to cover health care professionals’ costs in many instances.

In a previous study, Stewart et al^15^ assessed reimbursement amounts to health care professionals for vaccine administration and purchase under Medicaid FFS arrangements. Since 2012, the median reimbursement for HPV vaccine purchase has increased from $131.36 to $204.87 (likely owing primarily to introduction of the 9vHPV vaccine formulation [*CPT* code 90651] in lieu of the previous quadrivalent HPV vaccine). In contrast, median reimbursement for PPSV23 vaccine purchase (*CPT* code 90732) decreased from $130.27 in 2012 to $103.30 in 2019, providing further evidence that payments for some vaccines are not keeping pace with costs to health care professionals in all Medicaid programs. Financial concerns reduce health care professionals’ willingness to make vaccines available to adult patients^28,29^; Medicaid reimbursements that do not cover costs to health care professionals may thus reduce vaccination access for low-income adults.

Because Medicaid enrollee penetration varies by state, the effects of adult immunization policies on beneficiaries are disproportionate. Of the 10 largest Medicaid programs included in our survey (California, New York, Florida, Texas, Pennsylvania, Illinois, Ohio, Michigan, Washington, and Arizona), only 6 cover all ACIP-recommended adult immunizations, 5 with no cost sharing (California, New York, Illinois, Ohio, and Washington). However, while some of these programs grant complete vaccination coverage benefits, reimbursement for vaccine administration may be limited (California) or not permitted (Illinois), which may disincentivize health care professionals to vaccinate. Although Florida has the third-largest state Medicaid program in the country,^3^ adult vaccination coverage benefits are available only for adults aged 19 to 20 years or limited to pharmacy services for FFS recipients living in residential facilities. As Florida has not implemented Medicaid program expansion under the Patient Protection and Affordable Care Act, observed barriers to adult immunization services likely reduce vaccination coverage substantially.

### Limitations

Our study has several limitations. First, not all jurisdictions responded to our survey, so we obtained information about exact reimbursement amounts to health care professionals under MCO arrangements for only 87% of programs covering at least some beneficiaries through MCOs; this lack of response restricted our ability to effectively evaluate policies on reimbursement to health care professionals and adult vaccine access under MCO arrangements. Second, because some Medicaid programs did not participate, our results may not provide a complete picture of US adult vaccination benefits coverage policies. However, our results include the 10 largest Medicaid programs and comprise jurisdictions representing more than 93% of the Medicaid beneficiary population.^3^ Third, low-income adults may receive vaccination services outside of the Medicaid program, so our findings are an imperfect proxy for vaccine access in this population. Fourth, ACIP vaccine recommendations and Medicaid policies change frequently, so information reported by respondents may not be current.

## Conclusions

To our knowledge, this report presents the most comprehensive available examination of vaccination benefits coverage for low-income US adults and is the first to examine adult immunization coverage in both FFS and MCO arrangements. In many jurisdictions, adult Medicaid beneficiaries lack access to the full slate of ACIP-recommended vaccines. Even in programs providing complete vaccination coverage benefits, reimbursement amounts to health care professionals for vaccine purchase and administration may not fully cover costs to provide vaccination, disincentivizing health care professionals to vaccinate low-income adults. Increased vaccination coverage benefits parity across Medicaid programs and between traditionally eligible and expansion adult populations could decrease income-based health disparities and reduce the proportion of limited program funds expended to treat vaccine-preventable diseases.

## Notes

Supplement. **eTable 1.** Vaccine Benefits Coverage of All ACIP-Recommended Adult Vaccinations Under FFS Arrangements, by *CPT* Code **eTable 2.** Reimbursement Amounts to Health Care Professionals for Vaccine Purchase Under FFS Arrangements, by *CPT* Code
