# Supplementary material for: State Policies on Access to Vaccination Services for Low-Income Adults
Source: JAMA Netw Open. 2020 Apr 27;3(4):e203316. doi: 10.1001/jamanetworkopen.2020.3316 (PMC7186857; doi:10.1001/jamanetworkopen.2020.3316)
Supplement: Supplement. — eTable 1. Vaccine Benefits Coverage of All ACIP-Recommended Adult Vaccinations Under FFS Arrangements, by CPT Code eTable 2. Reimbursement Amounts to Health Care Professionals for Vaccine Purchase Under FFS Arrangements, by CPT Code [file jamanetwopen-3-e203316-s001.pdf]

Supplementary Online Content

Granade CJ, McCord RF, Bhatti AA, Lindley MC. State policies on access to vaccination services for low-income adults. *JAMA Netw Open.* 2020;3(4):e203316. doi:10.1001/jamanetworkopen.2020.3316

eTable 1. Vaccine Benefits Coverage of All ACIP-Recommended Adult Vaccinations Under FFS Arrangements, by *CPT* Code
eTable 2. Reimbursement Amounts to Health Care Professionals for Vaccine Purchase Under FFS Arrangements, by *CPT* Code

This supplementary material has been provided by the authors to give readers additional information about their work.

eTable 1. Vaccine Benefits Coverage of All ACIP-Recommended Adult Vaccinations Under FFS Arrangements, by *CPT* Code[a]

Programs	Influenza									MMR	Td
	90630	90654	90656	90658	90660	90661	90662	90672	90682	90707	90714
**Alabama**	0	1	1	1	1	1	1	0	1	1	1
**Alaska**	1	1	1	1	1	1	1	0	0	1	1
**Arizona**	1	0	1	1	1	0	1	1	1	1	1
**Arkansas**	1	1	1	1	1	0	1	1	1	1	1
**California**	1	1	1	1	1	0	1	0	1	1	1
**Colorado**	1	1	1	1	0	1	0	1	0	1	1
**Connecticut**	1	1	1	1	1	0	1	U	U	1	1
**Delaware**	1	1	1	1	1	1	1	1	1	1	1
**District of Columbia**	1	1	1	1	1	1	0	1	0	1	1
**Florida**[c]	0	0	1	1	0	0	0	0	0	1	1
**Georgia**	0	0	1	0	0	0	1	1	1	1	1
**Hawaii**[d]	see footnote	see footnote	see footnote	see footnote	see footnote	see footnote	see footnote	see footnote	see footnote	see footnote	see footnote
**Idaho**	1	1	1	1	1	1	1	1	1	1	1
**Illinois**	1	0	1	1	0	0	1	1	1	1	1
**Indiana**	1	1	1	1	1	1	1	1	1	1	1
**Iowa**	1	0	1	0	1	0	0	1	0	1	1
**Kansas**	1	1	1	1	0	1	1	1	1	1	1
**Kentucky**	0	1	1	1	1	1	1	1	1	1	1
**Louisiana**	0	1	1	1	0	0	0	1	1	1	1
**Maine**	0	0	1	1	1	0	1	1	1	1	1
**Maryland**	1	0	1	1	1	1	1	1	1	1	1
**Massachusetts**	0	1	0	1	1	1	0	0	1	1	0
**Michigan**	1	1	1	1	0	1	1	1	1	1	1
**Minnesota**	0	0	1	1	0	0	1	1	1	1	1
**Mississippi**	1	1	1	1	0	1	1	0	1	1	1

© 2020 Granade CJ et al. *JAMA Network Open.*

Programs	Influenza									MMR	Td
	90630	90654	90656	90658	90660	90661	90662	90672	90682	90707	90714
**Missouri**	1	1	1	1	1	0	0	1	0	1	1
**Montana**	1	0	1	0	0	0	0	0	1	1	1
**Nebraska**	1	1	1	1	1	0	1	1	1	1	1
**Nevada**	0	1	1	1	1	0	0	1	0	1	1
**New Hampshire**	1	1	1	1	1	1	1	U	U	1	1
**New Jersey**	1	1	1	1	1	1	1	U	U	1	1
**New Mexico**	1	1	1	1	1	1	1	1	1	1	1
**New York**	1	1	1	1	1	1	1	1	1	1	1
**North Carolina**	1	0	1	1	0	0	1	1	1	1	1
**North Dakota**	1	1	1	1	0	0	1	1	0	1	1
**Ohio**	1	1	1	1	1	0	1	1	1	1	1
**Oklahoma**	1	1	1	1	0	0	0	1	1	1	1
**Oregon**	1	1	1	1	1	1	1	1	1	1	1
**Pennsylvania**	1	1	1	1	1	1	0	1	1	1	1
**Rhode Island**	0	0	0	0	0	0	0	0	0	0	0
**South Carolina**	1	1	1	1	1	0	1	1	1	1	1
**South Dakota**	0	1	1	1	1	1	1	1	0	1	1
**Tennessee**[d]	see footnote	see footnote	see footnote	see footnote	see footnote	see footnote	see footnote	see footnote	see footnote	see footnote	see footnote
**Texas**	1	1	1	1	1	1	1	1	1	1	1
**Utah**	1	1	1	1	1	1	1	0	1	1	1
**Vermont**	1	1	1	1	0	1	1	1	1	1	1
**Virginia**	1	1	1	1	1	0	0	U	U	1	1
**Washington**	0	1	1	1	0	1	1	1	1	1	1
**West Virginia**	0	1	1	1	1	1	1	1	0	1	1
**Wisconsin**	0	0	1	1	1	1	1	1	0	1	1
**Wyoming**	0	0	1	1	1	0	0	1	1	1	1
**Total number of states**	34	36	47	45	33	26	35	36	33	48	47

© 2020 Granade CJ et al. *JAMA Network Open.*

Programs	Tdap	PCV13	PPSV23	Hep A	Hep A/B	Hep B				MenB	
	90715	90670	90732	90632	90636	90739	90740	90746	90747	90620	90621
Alabama	1	1	1	0	0	0	1	1	1	1	1
Alaska	1	0	1	1	1	0	1	1	0	0	0
Arizona	1	1	1	1	1	1	1	1	1	1	1
Arkansas	1	1	1	1	1	0	1	1	1	1	1
California	1	1	1	1	1	0	1	1	1	1	1
Colorado	1	1	1	1	1	1	1	1	1	1	1
Connecticut	1	1	1	1	1	1	1	1	1	1	1
Delaware	1	1	1	1	1	0	1	1	1	1	1
District of Columbia	1	1	1	1	1	0	1	1	1	1	1
Florida[c]	1	1	1	1	0	1	0	1	0	1	1
Georgia	1	1	1	1	1	1	0	1	1	1	1
Hawaii[d]	see footnote	see footnote	see footnote	see footnote	see footnote	see footnote	see footnote	see footnote	see footnote	see footnote	see footnote
Idaho	1	1	1	1	1	1	1	1	1	1	1
Illinois	1	1	1	1	1	1	1	1	1	1	1
Indiana	1	1	1	1	1	1	1	1	1	1	1
Iowa	1	1	1	1	1	0	1	1	0	1	1
Kansas	1	1	1	1	1	1	1	1	1	1	1
Kentucky	1	1	1	1	1	1	1	1	1	1	1
Louisiana	1	1	1	1	1	1	0	1	0	1	1
Maine	1	1	1	1	1	1	1	1	1	1	1
Maryland	1	1	1	1	1	1	1	1	1	1	1
Massachusetts	0	0	0	0	0	1	0	0	0	1	1
Michigan	1	1	1	1	1	1	1	1	1	1	1
Minnesota	1	1	1	1	1	1	1	1	1	1	1
Mississippi	1	1	1	0	0	0	1	1	1	1	1

© 2020 Granade CJ et al. *JAMA Network Open.*

Programs	Tdap	PCV13	PPSV23	Hep A	Hep A/B	Hep B				MenB	
	90715	90670	90732	90632	90636	90739	90740	90746	90747	90620	90621
**Missouri**	1	1	1	1	1	0	0	1	1	1	1
**Montana**	1	1	1	1	1	1	1	1	1	1	1
**Nebraska**	1	1	1	1	1	1	1	1	1	1	1
**Nevada**	1	1	1	1	1	0	1	1	1	0	0
**New Hampshire**	1	1	1	1	1	1	1	1	1	1	1
**New Jersey**	1	1	1	1	1	1	1	1	1	1	1
**New Mexico**	1	1	1	1	1	1	1	1	1	1	1
**New York**	1	1	1	1	1	1	1	1	1	1	1
**North Carolina**	1	1	1	1	1	1	1	1	1	1	1
**North Dakota**	1	1	1	0	1	0	1	1	1	1	1
**Ohio**	1	1	1	1	1	1	1	1	1	1	1
**Oklahoma**	1	1	1	1	1	1	1	1	1	0	0
**Oregon**	1	1	1	1	1	1	1	1	1	1	1
**Pennsylvania**	1	1	1	1	1	1	0	1	0	1	1
**Rhode Island**	1	0	1	1	1	0	1	1	1	0	0
**South Carolina**	1	1	1	1	1	1	1	1	1	1	1
**South Dakota**	1	1	1	1	1	0	1	1	1	1	1
**Tennessee**[d]	see footnote	see footnote	see footnote	see footnote	see footnote	see footnote	see footnote	see footnote	see footnote	see footnote	see footnote
**Texas**	1	1	1	1	1	0	1	1	1	1	1
**Utah**	1	1	1	1	1	1	1	1	1	1	1
**Vermont**	1	1	1	1	0	1	1	1	1	0	0
**Virginia**	1	1	1	1	1	1	1	1	1	1	1
**Washington**	1	1	1	1	1	1	1	1	1	1	1
**West Virginia**	1	1	1	1	1	1	1	1	1	1	1
**Wisconsin**	1	1	1	1	1	1	1	1	1	1	1
**Wyoming**	1	1	1	1	1	1	1	1	1	1	1
**Total number of states**	48	46	48	45	44	35	43	48	43	44	44

© 2020 Granade CJ et al. *JAMA Network Open.*

Programs	MenACWY		HPV		Varicella	Zoster		Hib		Total number of adult vaccine products covered	Total ACIP-recommended vaccines covered[b]
	90733	90734	4vHPV: 90649	9vHPV: 90651	90716	90736	90750	90647	90648		
**Alabama**	1	1	0	1	1	0	0	0	0	21	10
**Alaska**	1	1	1	1	1	1	1	0	0	22	10
**Arizona**	1	1	1	0	1	1	0	1	1	27	11
**Arkansas**	1	1	1	1	1	1	1	1	0	28	13
**California**	1	1	1	1	1	1	1	1	1	28	13
**Colorado**	1	1	1	1	1	1	1	1	0	27	13
**Connecticut**	1	1	1	1	1	1	1	U	U	26	12
**Delaware**	1	1	1	1	1	1	0	1	1	29	12
**District of Columbia**	1	1	1	1	1	1	0	1	1	27	12
**Florida[c]**	0	1	0	1	1	1	1	0	0	17	12
**Georgia**	0	1	0	1	1	1	1	0	0	21	12
**Hawaii[d]**	see footnote	see footnote	see footnote	see footnote	see footnote	see footnote	see footnote	see footnote	see footnote	see footnote	see footnote
**Idaho**	1	1	1	1	1	1	0	1	1	30	12
**Illinois**	1	1	1	1	1	1	1	1	1	28	13
**Indiana**	1	1	1	1	1	1	1	1	1	31	13
**Iowa**	1	1	1	1	1	1	1	1	1	24	13
**Kansas**	1	1	0	1	1	0	1	1	1	28	13
**Kentucky**	1	1	1	0	1	1	1	1	1	29	12
**Louisiana**	0	1	1	1	1	1	1	1	1	24	13
**Maine**	1	1	1	1	1	1	1	1	1	28	13
**Maryland**	1	1	0	1	1	1	1	1	1	29	13
**Massachusetts**	1	1	1	1	1	1	1	1	1	18	9
**Michigan**	0	1	0	1	1	1	1	0	0	26	12
**Minnesota**	0	1	1	1	1	1	1	0	1	25	13

© 2020 Granade CJ et al. *JAMA Network Open.*

Programs	MenACWY		HPV		Varicella	Zoster		Hib		Total number of adult vaccine products covered	Total ACIP-recommended vaccines covered[b]
	90733	90734	4vHPV: 90649	9vHPV: 90651	90716	90736	90750	90647	90648		
**Mississippi**	1	1	1	1	1	1	0	1	1	25	11
**Missouri**	0	1	1	1	1	1	1	1	1	25	13
**Montana**	0	1	0	1	1	1	1	0	0	21	12
**Nebraska**	1	1	1	1	1	0	0	0	1	27	12
**Nevada**	1	1	1	0	1	1	0	1	1	22	10
**New Hampshire**	1	1	1	1	1	1	1	1	1	29	13
**New Jersey**	1	1	1	1	1	1	0	U	U	26	11
**New Mexico**	1	1	1	1	1	1	1	1	1	31	13
**New York**	1	1	1	1	1	1	1	1	1	31	13
**North Carolina**	1	1	1	1	1	1	1	1	1	28	13
**North Dakota**	1	1	1	1	1	1	0	0	0	23	11
**Ohio**	1	1	1	1	1	1	1	1	1	30	13
**Oklahoma**	1	0	0	1	1	1	1	0	0	22	11
**Oregon**	1	1	1	1	1	1	1	1	1	31	13
**Pennsylvania**	1	1	1	1	1	1	1	1	1	28	13
**Rhode Island**	1	0	1	0	0	1	0	0	0	10	5
**South Carolina**	1	1	0	0	1	0	0	1	1	26	11
**South Dakota**	1	1	1	1	1	1	0	1	1	27	12
**Tennessee[d]**	see footnote	see footnote	see footnote	see footnote	see footnote	see footnote	see footnote	see footnote	see footnote	see footnote	see footnote
**Texas**	1	1	0	1	1	0	0	0	1	26	12
**Utah**	1	1	1	1	1	1	1	1	1	30	13
**Vermont**	1	0	0	0	1	1	0	1	1	23	10
**Virginia**	1	1	1	1	1	0	0	U	U	23	11
**Washington**	1	1	0	1	1	1	1	1	1	28	13
**West Virginia**	1	1	1	1	1	1	1	1	1	29	13

© 2020 Granade CJ et al. *JAMA Network Open*.

Programs	MenACWY		HPV		Varicella	Zoster		Hib		Total number of adult vaccine products covered	Total ACIP-recommended vaccines covered[b]
	90733	90734	4vHPV: 90649	9vHPV: 90651	90716	90736	90750	90647	90648		
**Wisconsin**	1	1	1	1	1	1	1	1	1	28	13
**Wyoming**	1	1	1	1	1	1	1	1	1	27	13
**Total number of states**	42	46	37	43	48	43	33	34	35		

Abbreviations: 4vHPV = 4-valent human papillomavirus vaccine; 9vHPV = 9-valent human papillomavirus vaccine; ACIP = Advisory Committee on Immunization Practices; CPT code = Current Procedural Terminology code; FFS = fee-for-service; HepA = hepatitis A vaccine; HepA/B = hepatitis A and B combination vaccine; HepB = hepatitis B vaccine; Hib = *Haemophilus influenzae* type b vaccine; MenACWY = serogroup A, C, W, and Y meningococcal vaccine; MenB = serogroup B meningococcal vaccine; MMR = measles, mumps, rubella vaccine; PCV13 = pneumococcal conjugate vaccine; PPSV23 = pneumococcal polysaccharide vaccine; Tdap = tetanus toxoid, reduced diphtheria toxoid, and acellular pertussis vaccine; U = Unable to determine.

[a] Data from public domain document review. Because the following Medicaid programs did not participate in the semi-structed survey, data from Arkansas, Iowa, New Jersey, North Carolina, and Utah were not validated.

[b] There are multiple vaccine products available for hepatitis B, serogroup A, C, W, and Y meningococcal, serogroup B meningococcal, and *Haemophilus influenzae* type b vaccines. Therefore, ACIP coverage was determined using the most frequently available CPT code for each of these vaccines.

[c] In Florida, adult vaccination coverage benefits for influenza, pneumococcal, and zoster immunization apply to vaccinations given through the pharmacy to adult beneficiaries who reside in nursing facilities. All other vaccines are covered for adults aged 19-20 years only.

[d] Both Hawaii and Tennessee Medicaid are under 100% managed care organization arrangements.

© 2020 Granade CJ et al. *JAMA Network Open.*

eTable 2. Reimbursement Amounts to Health Care Professionals for Vaccine Purchase Under FFS Arrangements, by *CPT* Code[a,b,c]

Programs	Influenza									MMR	Td
	**90630[d]**	**90654**	**90656**	**90658[e]**	**90660[f]**	**90661[d]**	**90662**	**90672**	**90682**	**90707**	**90714[d]**
**Alabama**	NC	18.92	19.25	10.80	20.99	22.29	49.03	NC	53.37	70.92	22.07
**Alaska**	By report	16.74	15.56	15.56	22.03	13.35	13.70	NC	NC	54.69	8.00
**Arizona**	19.32	NC	18.29	10.54	20.91	NC	46.58	24.60	46.31	55.87	22.96
**Arkansas**	25.07	10.31	13.12	12.06	18.40	NC	13.80	25.54	46.31	34.64	14.00
**California**	24.80	20.36	24.23	22.70	17.88	NC	57.83	NC	57.83	73.13	27.61
**Colorado**	16.54	19.70	17.18	15.80	NC	14.82	NC	22.43	NC	70.92	32.89
**Connecticut**	20.34	MP	19.25	MP	MP	NC	49.03	U	U	MP	25.80
**Delaware**	MP	MP	16.72	15.87	MP	MP	MP	MP	MP	MP	MP
**District of Columbia**	16.27	15.13	15.40	16.28	55.20	17.72	NC	21.50	NC	34.91	20.64
**Florida**	NC	NC	16.33	16.33	NC	NC	NC	NC	NC	75.04	32.89
**Georgia**	NC	NC	19.77	NC	NC	NC	53.37	22.95	53.37	75.04	23.14
**Hawaii[g]**	see footnote	see footnote	see footnote	see footnote	see footnote	see footnote	see footnote	see footnote	see footnote	see footnote	see footnote
**Idaho**	18.31	17.03	17.79	13.22	21.11	20.06	48.03	24.19	48.03	70.19	23.22
**Illinois**	20.71	NC	19.77	17.74	NC	NC	53.37	22.95	53.37	70.92	23.15
**Indiana**	18.08	16.77	17.56	16.14	0.00	19.87	48.50	24.10	48.50	76.43	18.89
**Iowa**	17.10	NC	12.91	NC	28.91	NC	NC	23.00	NC	50.50	40.24
**Kansas**	20.34	18.92	19.77	20.16	NC	19.68	53.37	23.70	46.31	70.92	23.92
**Kentucky**	NC	18.92	12.40	14.35	21.70	20.66	31.82	24.60	46.31	56.14	19.30
**Louisiana**	NC	17.22	19.77	15.37	NC	NC	NC	25.00	53.37	75.04	23.13
**Maine**	NC	NC	19.77	11.37	23.46	NC	53.37	26.88	53.37	47.07	23.13
**Maryland**	20.34	NC	19.78	18.24	22.32	By report	53.38	26.88	53.38	47.01	23.15
**Massachusetts**	NC	IC	NC	IC	IC	IC	NC	NC	IC	IC	NC
**Michigan**	20.34	18.92	19.77	17.72	NC	22.29	53.37	24.32	53.37	77.15	23.14
**Minnesota**	NC	NC	19.77	16.12	NC	NC	53.37	26.87	53.37	75.04	23.12
**Mississippi**	20.34	18.92	19.77	37.23	NC	22.29	53.37	NC	53.37	84.65	23.14

© 2020 Granade CJ et al. *JAMA Network Open.*

Programs	Influenza									MMR	Td
	90630[d]	90654	90656	90658[e]	90660[f]	90661[d]	90662	90672	90682	90707	90714[d]
**Missouri**	0.00	0.00	7.11	13.51	21.97	NC	NC	5.27	NC	56.04	15.80
**Montana**	18.31	NC	19.77	NC	NC	NC	NC	NC	53.37	75.04	23.14
**Nebraska**	19.19	17.86	19.77	17.22	10.71	NC	53.37	24.51	46.31	80.14	23.13
**Nevada**	NC	18.60	13.48	13.74	9.34	NC	NC	9.34	NC	33.23	20.43
**New Hampshire**	20.12	19.91	13.68	5.38	57.50	19.01	19.01	20.11	35.28	71.49	22.50
**New Jersey**	22.26	18.00	32.19	32.19	25.69	20.70	32.50	U	U	62.33	26.05
**New Mexico**	20.34	18.92	19.77	11.37	23.46	22.29	53.37	26.88	53.37	47.07	23.13
**New York**	MP	MP	MP	MP	MP	MP	MP	MP	MP	MP	MP
**North Carolina**	17.74	NC	16.75	15.33	NC	NC	47.58	23.64	47.58	74.97	32.33
**North Dakota**	20.34	18.92	17.72	12.68	NC	NC	42.72	26.88	NC	67.03	23.23
**Ohio**	20.34	18.92	19.77	10.39	23.46	NC	53.37	26.88	53.37	57.66	23.13
**Oklahoma**	19.68	18.30	19.13	10.65	NC	NC	NC	22.20	51.64	51.08	22.37
**Oregon**	20.34	18.92	19.77	15.83	23.46	22.29	53.37	26.88	53.37	70.85	23.13
**Pennsylvania[h]**	see footnote	see footnote	see footnote	see footnote	see footnote	see footnote	NC	see footnote	see footnote	see footnote	see footnote
**Rhode Island**	NC	NC	NC	NC	NC	NC	NC	NC	NC	NC	NC
**South Carolina**	17.56	16.33	17.06	15.74	20.25	NC	46.07	22.58	46.07	69.41	23.55
**South Dakota**	NC	18.93	16.27	13.83	28.29	22.30	39.22	24.68	NC	68.38	37.66
**Tennessee[g]**	see footnote	see footnote	see footnote	see footnote	see footnote	see footnote	see footnote	see footnote	see footnote	see footnote	see footnote
**Texas**	19.17	22.96	17.69	35.86	24.65	42.95	47.75	24.65	47.75	75.76	20.71
**Utah**	21.41	13.52	19.77	15.64	22.38	% of charges	53.37	NC	53.37	75.04	23.14
**Vermont**	20.34	17.60	19.18	11.36	NC	20.73	51.77	24.99	51.77	34.93	22.45
**Virginia**	20.34	18.38	19.24	13.22	22.32	NC	NC	U	U	70.92	32.89
**Washington**	NC	DC	19.77	33.24	NC	31.28	53.37	22.95	53.37	70.92	23.13

© 2020 Granade CJ et al. *JAMA Network Open.*

Programs	Influenza									MMR	Td
	**90630[d]**	**90654**	**90656**	**90658[e]**	**90660[f]**	**90661[d]**	**90662**	**90672**	**90682**	**90707**	**90714[d]**
**West Virginia**	NC	Carrier-priced	Carrier-priced	Carrier-priced	Carrier-priced	Carrier-priced	Carrier-priced	Carrier-priced	NC	Carrier-priced	Carrier-priced
**Wisconsin**	NC	NC	17.19	15.39	26.77	25.60	34.18	30.19	NC	66.10	21.06
**Wyoming**	NC	NC	17.72	14.41	23.46	NC	NC	26.88	46.31	44.40	21.91
**Median**	20.34	18.60	19.16	15.48	22.32	20.73	50.40	24.51	53.37	70.52	23.13
**CDC price**	12.38	U	U	N/A	N/A	15.28	U	18.88	U	49.54	18.32
**Private sector price**	16.82	U	U	N/A	N/A	23.40	U	23.70	U	75.04	29.48

Programs	Tdap	PCV13	PPSV23	Hep A	Hep A/B	Hep B				MenB	
	**90715[d]**	**90670**	**90732**	**90632[d]**	**90636**	**90739**	**90740**	**90746[d]**	**90747**	**90620**	**90621**
**Alabama**	31.67	192.64	98.85	NC	NC	NC	126.60	63.30	126.60	160.75	122.65
**Alaska**	40.10	NC	55.82	71.07	100.71	NC	188.43	58.99	NC	NC	NC
**Arizona**	30.66	194.85	102.36	44.94	89.39	124.55	123.74	61.86	123.74	115.50	112.04
**Arkansas**	28.80	9.56	12.34	61.05	91.28	NC	153.14	57.58	110.20	183.11	131.81
**California**	35.36	207.57	112.21	63.10	103.96	NC	134.71	69.58	134.71	169.46	137.33
**Colorado**	44.24	180.05	94.51	66.91	101.00	131.10	55.65	55.65	76.43	165.75	133.62
**Connecticut**	30.96	192.64	107.75	51.38	MP	131.10	126.60	63.30	126.60	MP	MP
**Delaware**	MP	MP	MP	MP	MP	NC	MP	MP	MP	MP	MP
**District of Columbia**	24.77	154.11	86.20	41.11	78.16	NC	101.28	50.64	101.28	153.09	115.75
**Florida**	41.39	180.05	100.19	67.55	NC	115.00	NC	57.25	NC	165.75	133.62
**Georgia**	32.05	215.33	114.21	59.65	103.75	131.10	NC	67.06	134.12	170.63	140.15
**Hawaii[g]**	see footnote	see footnote	see footnote	see footnote	see footnote	see footnote	see footnote	see footnote	see footnote	see footnote	see footnote
**Idaho**	27.86	173.37	96.97	46.24	85.00	117.99	113.94	56.97	113.94	149.06	120.50
**Illinois**	30.90	205.11	107.75	58.64	101.00	131.10	130.25	65.12	130.24	165.75	133.62
**Indiana**	39.69	188.26	105.19	70.84	101.43	120.75	173.55	59.33	118.65	139.51	139.51
**Iowa**	40.24	112.56	56.56	72.59	87.35	NC	182.95	52.78	NC	153.76	112.75
**Kansas**	42.14	215.33	94.51	66.28	101.64	131.10	134.12	67.06	134.12	165.75	133.62
**Kentucky**	31.84	145.11	72.35	51.55	92.50	117.99	119.42	59.71	119.42	122.95	95.75

© 2020 Granade CJ et al. *JAMA Network Open.*

Programs	Tdap[d]	PCV13	PPSV23	Hep A	Hep A/B	HepB				MenB	
	90715	90670	90732	90632[d]	90636	90739	90740	90746[d]	90747	90620	90621
**Louisiana**	32.34	205.11	107.75	58.27	102.50	131.10	NC	65.12	NC	160.00	132.87
**Maine**	32.34	205.11	107.75	58.27	110.64	131.10	130.25	65.12	130.25	147.37	122.70
**Maryland**	30.90	205.12	107.75	58.65	78.16	131.10	130.25	65.13	130.25	160.75	115.75
**Massachusetts**	NC	NC	NC	NC	NC	IC	NC	NC	NC	IC	IC
**Michigan**	32.05	215.33	114.21	59.65	108.65	131.10	134.12	67.06	134.12	180.20	140.84
**Minnesota**	32.05	215.33	114.21	59.65	104.00	131.10	134.12	67.06	134.12	170.75	140.27
**Mississippi**	32.05	215.33	114.21	NC	NC	NC	134.12	67.06	134.12	198.75	160.20
**Missouri**	21.74	158.83	11.33	69.55	96.35	NC	NC	54.12	43.38	5.27	5.27
**Montana**	32.05	215.33	114.21	59.65	101.00	131.10	134.12	67.06	134.12	165.75	133.62
**Nebraska**	32.33	205.11	107.75	58.27	107.87	131.10	130.24	65.12	130.24	177.02	142.71
**Nevada**	35.65	139.49	11.12	56.31	62.71	NC	102.77	51.08	102.77	NC	NC
**New Hampshire**	46.23	136.14	95.27	61.00	123.00	115.92	197.72	65.00	110.20	160.64	115.46
**New Jersey**	47.25	132.10	35.76	80.95	103.04	By report	209.86	65.25	209.86	158.40	113.85
**New Mexico**	32.34	205.11	107.75	58.27	113.28	131.10	130.25	65.12	130.25	169.60	121.9
**New York**	MP	MP	MP	MP	MP	MP	MP	MP	MP	MP	MP
**North Carolina**	40.11	200.00	105.17	70.87	105.58	118.45	170.25	59.95	119.89	175.10	143.70
**North Dakota**	29.98	181.06	89.95	NC	92.50	NC	122.96	61.48	122.96	160.75	122.65
**Ohio**	32.34	205.11	107.75	58.27	97.37	131.10	130.25	65.12	130.25	171.20	123.05
**Oklahoma**	32.34	198.45	104.24	56.38	82.50	126.84	126.01	63.01	126.01	NC	NC
**Oregon**	32.34	205.11	107.75	58.27	78.30	131.10	130.25	65.12	130.25	146.75	119.01
**Pennsylvania[h]**	see footnote	see footnote	see footnote	see footnote	see footnote	see footnote	NC	see footnote	NC	see footnote	see footnote
**Rhode Island**	32.34	NC	107.75	58.27	94.73	NC	130.25	65.12	130.25	NC	NC
**South Carolina**	39.04	177.05	93.00	65.71	99.14	113.16	163.26	56.21	112.42	162.98	131.36
**South Dakota**	41.83	172.50	88.45	65.77	94.36	NC	112.88	57.45	112.88	163.98	163.98
**Tennessee[g]**	see footnote	see footnote	see footnote	see footnote	see footnote	see footnote	see footnote	see footnote	see footnote	see footnote	see footnote

© 2020 Granade CJ et al. *JAMA Network Open.*

Programs	Tdap[d]	PCV13	PPSV23	Hep A	Hep A/B	HepB				MenB	
	90715	90670	90732	90632[d]	90636	90739	90740	90746[d]	90747	90620	90621
Texas	27.65	183.52	96.41	52.47	108.21	NC	116.54	58.27	116.54	177.88	95.59
Utah	32.05	215.33	114.21	59.65	98.10	131.10	134.12	67.06	134.12	170.75	140.26
Vermont	29.97	198.96	104.51	45.88	NC	127.17	126.34	63.17	126.34	NC	NC
Virginia	40.05	180.05	94.51	58.64	77.41	131.10	130.24	63.29	130.24	160.75	133.62
Washington	32.05	215.33	114.21	59.65	98.10	131.10	134.12	67.02	134.12	165.75	133.62
West Virginia	Carrier-priced	Carrier-priced	Carrier-priced	Carrier-priced	Carrier-priced	Carrier-priced	Carrier-priced	Carrier-priced	Carrier-priced	Carrier-priced	Carrier-priced
Wisconsin	31.58	176.46	90.82	52.71	95.81	115.00	122.73	63.02	122.73	164.06	119.06
Wyoming	28.29	181.06	89.95	49.73	95.23	115.00	122.96	61.47	122.96	160.75	122.65
Median	32.19	193.75	103.30	58.65	98.10	131.10	130.25	63.30	126.60	164.02	131.59
CDC price	24.80	125.07	66.20	30.76	61.86	69.75	U	25.18	U	103.94	85.10
Private sector price	43.35	188.26	100.19	68.90	104.00	115.75	U	41.45	U	170.75	139.52

Programs	MenACWY		HPV		Varicella	Zoster[i]		Hib	
	90733[f]	90734[d]	4vHPV: 90649[f]	9vHPV: 90651	90716	90736	90750	90647	90648[d]
Alabama	120.83	120.83	NC	204.87	112.94	NC	NC	NC	NC
Alaska	119.31	117.03	142.50	245.00	95.49	172.06	By report	NC	NC
Arizona	101.17	113.66	95.07	NC	94.27	206.07	NC	19.78	5.30
Arkansas	69.85	65.60	9.56	9.56	51.53	129.20	112.00	25.09	NC
California	126.88	121.33	164.72	208.58	125.73	218.97	144.46	29.94	20.68
Colorado	124.44	116.30	167.94	204.87	122.02	212.67	140.00	26.23	NC
Connecticut	MP	MP	MP	MP	MP	MP	MP	U	U
Delaware	MP	MP	MP	158.41	MP	MP	NC	MP	MP
District of Columbia	85.19	82.00	130.27	162.45	39.94	114.69	NC	20.37	15.75
Florida	NC	126.95	NC	217.11	129.30	212.67	144.20	NC	NC
Georgia	NC	122.18	NC	216.99	129.30	212.67	144.20	NC	NC
Hawaii[g]	see footnote	see footnote	see footnote	see footnote	see footnote	see footnote	see footnote	see footnote	see footnote

© 2020 Granade CJ et al. *JAMA Network Open.*

Programs	MenACWY		HPV		Varicella	Zoster[i]		Hib	
	90733[f]	90734[d]	4vHPV: 90649[f]	9vHPV: 90651	90716	90736	90750	90647	90648[d]
Idaho	95.84	90.20	131.73	216.81	77.33	159.16	NC	23.34	21.78
Illinois	123.17	126.95	161.69	204.87	122.02	223.12	140.00	26.23	16.05
Indiana	126.08	102.50	167.39	227.18	117.80	161.63	151.41	26.76	10.61
Iowa	107.55	120.32	145.36	140.45	87.87	162.36	141.85	20.91	22.52
Kansas	106.49	121.63	NC	204.87	122.02	NC	140.00	26.12	24.34
Kentucky	106.49	117.41	141.38	NC	94.14	165.69	280.00	22.77	26.21
Louisiana	NC	116.39	121.03	217.71	129.30	212.67	140.00	16.15	10.55
Maine	106.49	97.49	120.00	172.04	81.67	187.66	148.95	22.16	23.41
Maryland	100.43	82.00	NC	177.70	81.75	212.00	140.00	20.37	22.83
Massachusetts	IC	IC	IC	IC	IC	IC	IC	IC	IC
Michigan	NC	128.85	NC	229.34	136.26	236.51	148.40	NC	NC
Minnesota	NC	122.31	160.17	217.11	129.30	212.67	144.20	NC	10.85
Mississippi	0.00	0.00	384.90	491.38	146.28	267.74	NC	62.66	18.75
Missouri	NC	120.01	152.84	5.27	89.09	162.25	164.56	27.48	5.27
Montana	NC	126.95	NC	217.11	129.30	223.12	144.20	NC	NC
Nebraska	131.55	124.21	171.06	231.87	10.71	NC	NC	NC	11.59
New Hampshire	113.76	111.61	148.49	165.17	122.51	198.71	208.24	0.01	27.49
New Jersey	127.85	125.46	165.49	163.97	105.50	188.66	NC	U	U
New Mexico	106.49	127.15	130.00	176.95	81.67	201.88	MP	20.37	23.34
New York	MP	MP	MP	MP	MP	MP	MP	MP	MP
North Carolina	123.68	133.90	165.77	222.85	132.40	229.81	148.53	26.25	10.40
North Dakota	117.42	112.93	160.17	193.63	115.16	196.91	NC	NC	NC
Ohio	126.39	115.88	144.43	173.70	104.85	210.93	149.80	23.56	28.11
Oklahoma	103.03	NC	NC	165.86	88.75	171.88	165.45	NC	NC
Oregon	123.17	111.84	153.79	196.70	121.90	197.49	138.60	26.21	10.45
Pennsylvania[h]	see footnote	see footnote	see footnote	see footnote	see footnote	see footnote	see footnote	see footnote	see footnote

© 2020 Granade CJ et al. *JAMA Network Open.*

Programs	MenACWY		HPV		Varicella	Zoster[i]		Hib	
	90733[f]	90734[d]	4vHPV: 90649[f]	9vHPV: 90651	90716	90736	90750	90647	90648[d]
**Rhode Island**	106.49	NC	135.66	NC	NC	152.00	NC	NC	NC
**South Carolina**	104.66	114.32	NC	NC	119.94	NC	NC	25.69	10.26
**South Dakota**	110.15	118.71	166.16	197.52	117.47	216.94	NC	31.55	17.42
**Tennessee[g]**	see footnote	see footnote	see footnote	see footnote	see footnote	see footnote	see footnote	see footnote	see footnote
**Texas**	132.15	124.77	NC	193.64	130.91	NC	NC	NC	17.10
**Utah**	147.65	122.31	280.03	217.11	112.94	138.23	144.20	26.23	10.85
**Vermont**	58.66	NC	NC	NC	57.86	114.69	NC	28.00	28.00
**Virginia**	106.49	116.30	204.87	204.87	122.02	NC	NC	U	U
**Washington**	116.30	126.95	NC	204.87	122.02	212.67	140.00	78.70	16.05
**West Virginia**	Carrier-priced	Carrier-priced	Carrier-priced	Carrier-priced	Carrier-priced	Carrier-priced	Carrier-priced	Carrier-priced	Carrier-priced
**Wisconsin**	109.80	118.48	163.48	204.12	110.98	169.00	140.00	26.96	30.80
**Wyoming**	100.46	112.93	160.17	193.63	77.04	196.91	140.00	20.91	22.08
**Median**	108.68	117.95	153.79	204.87	114.05	197.49	144.20	25.69	18.09
**CDC price**	N/A	71.34	N/A	144.99	84.88	134.16	102.61	13.21	9.47
**Private sector price**	N/A	126.53	N/A	217.11	129.30	212.67	144.20	26.23	13.68

Abbreviations: 4-valent human papillomavirus vaccine; 9vHPV = 9-valent human papillomavirus vaccine; ACIP = Advisory Committee on Immunization Practices; CPT code = Current Procedural Terminology code; DC = Different code; FFS = fee-for-service; HepA = hepatitis A vaccine; HepA/B = hepatitis A and B combination vaccine; HepB = hepatitis B vaccine; Hib = *Haemophilus influenzae* type b vaccine; IC = individual consideration; MenACWY = serogroup A, C, W, and Y meningococcal vaccine; MenB = serogroup B meningococcal vaccine; MMR = measles, mumps, rubella vaccine; MP = Manually priced; N/A = Not applicable; NC = Not covered; PCV13 = pneumococcal conjugate vaccine; PPSV23 = pneumococcal polysaccharide vaccine; Tdap = tetanus toxoid, reduced diphtheria toxoid, and acellular pertussis vaccine; U = Unable to determine.

[a] Data from public domain document review with reimbursement amounts shown in 2018 US dollars. Because the following Medicaid programs did not participate in the semi-structed survey, data from Arkansas, Iowa, New Jersey, North Carolina, and Utah were not validated.

[b] Data on reimbursement to health care professionals for adult vaccine purchase current as of 4/30/2019.

© 2020 Granade CJ et al. *JAMA Network Open.*

[c] Cost data for CDC and manufacturer-reported prices current as of 8/01/2019.
[d] There are multiple vaccine products available for CPT codes 90630 (influenza), 90632 (HepA), 90648 (Hib), 90661(influenza), 90734 (MenACWY), 90746 (HepB), 90714 (Td), and 90715 (Tdap). Therefore, the CDC and manufacturer-reported prices were averaged for each code and the average is presented here.
[e] For CPT code 90658 (influenza), the CDC and manufacturer-reported prices are marked "N/A" because the CPT code is no longer valid.
[f] For CPT codes 90660 (influenza), 90733 (MenACWY), and 90649 (4vHPV), the CDC and manufacturer-reported prices are marked "N/A" because the vaccine is no longer in production.
[g] Both Hawaii and Tennessee are under 100% managed care organization arrangements.
[h] Pennsylvania reimburses adult vaccine purchase for actual dose administered by using each vaccine's associated national drug code (NDC).
[i] For 90736 (zoster), this information is as of 6/01/2018.

© 2020 Granade CJ et al. *JAMA Network Open.*
